# Repetitive Transcranial Magnetic Stimulation for Major Depressive Disorder: Clinical Evidence, Neurobiological Mechanisms, and Treatment Perspectives

**DOI:** 10.7759/cureus.108454

**Published:** 2026-05-07

**Authors:** Diego A Angulo Rodríguez, Maria F Gonzalez Espinosa, Gerson Olivera Morales, Natalia M Mora, Fernando E Medina

**Affiliations:** 1 General Medicine, Private Practice, Heredia, CRI; 2 General Medicine, Private Practice, San Jose, CRI; 3 Hemodynamics, Hospital San Rafael de Alajuela, Heredia, CRI; 4 Surgery, Caja Costarricense del Seguro Social, San Jose, CRI

**Keywords:** biomarkers, brain stimulation, dlpfc, functional connectivity, major depressive disorder, neuromodulation therapy, repetitive transcranial magnetic stimulation, theta burst stimulation, treatment-resistant depression

## Abstract

Major depressive disorder (MDD) remains one of the leading causes of disability worldwide, and a substantial proportion of patients do not achieve remission with standard pharmacological treatments. In this context, repetitive transcranial magnetic stimulation (rTMS) has gained increasing clinical recognition as a non-invasive alternative with a growing evidence base.

We conducted a narrative review through a structured PubMed search combining the terms "major depressive disorder," "repetitive transcranial magnetic stimulation," "rTMS," "theta burst stimulation," and "treatment-resistant depression." We prioritized randomized controlled trials, meta-analyses, and international clinical guidelines published between 2006 and 2025, favoring sham-controlled and multisite designs. A total of 31 articles were included.

The available evidence supports the use of rTMS in patients with MDD who have not responded adequately to antidepressant therapy, though response rates remain modest--approximately 29% for high-frequency protocols in controlled trials. No reliable method currently exists for predicting which patients will benefit before treatment begins. Theta burst stimulation has shown comparable efficacy to conventional protocols in shorter sessions, and accelerated paradigms, such as Stanford Neuromodulation Therapy, have yielded striking preliminary results, though replication in larger and more representative samples is still needed. Neuroimaging biomarkers, particularly resting-state connectivity measures, show promise but remain outside routine clinical reach. How to sustain response after an acute course--and for whom--remains an open and underexplored question.

rTMS has earned its place in the treatment of MDD. What the field still needs to answer is who benefits most, when to introduce it, and how to keep patients well over time.

## Introduction and background

The pharmacological management of major depressive disorder (MDD) has long relied on monoaminergic agents as a first-line approach, yet the limitations of this strategy have become increasingly apparent. Data from large pragmatic trials, including the STAR*D study, indicate that fewer than one-third of patients achieve remission after an initial antidepressant trial, and that cumulative remission rates decline progressively with each subsequent treatment attempt [1]. This therapeutic gap, affecting a substantial proportion of patients who fail to achieve adequate response despite multiple pharmacological trials, has driven renewed interest in non-pharmacological and neuromodulatory approaches.

Repetitive transcranial magnetic stimulation (rTMS) has emerged as a non-invasive brain stimulation technique with growing evidence supporting its use in the treatment of depression. Over the last 10 years, the use of standardized stimulation settings and expert consensus guidelines has made rTMS more consistent and safer to apply. Nevertheless, response rates remain modest, and the ability to predict which patients will benefit before treatment begins remains limited. Current clinical guidelines recognize rTMS as an evidence-based option for patients with insufficient response to antidepressant therapy, particularly in moderate to severe non-psychotic depression [2,3].

While rTMS is effective, it is often positioned as a later-stage intervention after multiple pharmacological treatment failures. However, new findings indicate that rTMS could be used more flexibly, depending on factors such as symptom severity, degree of treatment resistance, comorbid conditions, and patient preference [3,4]. That said, evidence supporting earlier use of rTMS remains limited, and the optimal sequencing of rTMS within treatment algorithms has not been established by prospective trials. In addition, advances in neuroimaging have provided greater insight into the neural circuits modulated by rTMS, particularly fronto-limbic networks involved in mood regulation.

This narrative review aims to integrate current clinical evidence and neurobiological mechanisms of rTMS in MDD, with particular emphasis on its role within treatment algorithms, practical clinical considerations, safety profile, and future directions. A narrative approach was chosen, given the broad and multidimensional scope of the topic, which encompasses mechanistic, clinical, and practical dimensions that do not lend themselves to a single structured research question.

## Review

Methods

This paper is a narrative review synthesizing clinical and neurobiological evidence on rTMS in MDD. We chose a narrative approach because the topic spans mechanistic, clinical, and practical dimensions that do not lend themselves to a single structured research question.

We searched PubMed using the following key terms: "major depressive disorder," "repetitive transcranial magnetic stimulation," "rTMS," "theta burst stimulation," and "treatment-resistant depression." The search was restricted to PubMed, and we acknowledge this as a limitation, since studies indexed exclusively in other databases may not have been captured.

We prioritized randomized controlled trials, meta-analyses, and international clinical guidelines because of their direct relevance to clinical practice. Neurobiological and neuroimaging studies were included when they helped explain the mechanisms underlying rTMS effects at the circuit and network levels.

We included studies involving adult patients with MDD that evaluated rTMS, including conventional rTMS, theta burst stimulation, and deep TMS protocols, and were published in English between 2006 and 2025. The 2006 lower limit was chosen to focus on the contemporary evidence base, coinciding with the period in which rTMS research began producing large-scale randomized controlled trials and formal clinical guidelines. We excluded studies unrelated to depressive disorders, case reports or very small uncontrolled samples, and articles without clear clinical or mechanistic relevance.

No formal quality assessment tool was used, and no PRISMA flow diagram was generated, consistent with the narrative design. We aimed to provide a clinically oriented synthesis rather than a systematic or quantitative appraisal of the evidence.

Discussion

rTMS in Treatment Algorithms for MDD

rTMS is currently recognized as an evidence-based intervention for MDD, particularly in patients who have not achieved an adequate response to antidepressant therapy [3,5]. Contemporary clinical guidelines position rTMS as a therapeutic option following insufficient response to at least one or two adequate pharmacological trials, depending on symptom severity and treatment history. Although no universal consensus exists on the precise definition of treatment-resistant depression, the most widely used criterion requires failure to respond to at least two antidepressant trials at a therapeutic dose and for an adequate duration [3]. What constitutes an 'adequate' trial is not uniformly defined across guidelines or clinical practice, reflecting an ongoing limitation in the field [2,3]. In cases of moderate to severe non-psychotic depression, rTMS may be considered before more invasive procedures, especially in outpatient settings [2].

The clinical evidence supporting this recommendation rests on a series of well-designed randomized controlled trials conducted over the past two decades. In the pivotal multisite trial by O'Reardon and colleagues, active high-frequency rTMS applied to the left dorsolateral prefrontal cortex (DLPFC) produced significantly greater reductions in depressive symptoms compared with sham stimulation, with response rates of approximately 24% in the active group versus 11% in the sham group at the end of acute treatment, assessed in patients with MDD who had not responded to prior antidepressant therapy [5]. These findings were subsequently reinforced by a large sham-controlled trial conducted by George and colleagues, which enrolled 190 patients who had failed at least one antidepressant trial and demonstrated comparable response patterns under more stringent enrollment criteria [6]. The FDA cleared the first TMS device for MDD in 2008, drawing directly on data from these two trials--a decision that marked a turning point in how treatment-resistant depression is managed in outpatient psychiatry.

Compared with electroconvulsive therapy (ECT), rTMS may offer important advantages in terms of tolerability and cognitive safety, although ECT remains the more established option for severe or psychotic depression [7]. Unlike ECT, rTMS does not require anesthesia and is associated with minimal cognitive adverse effects, making it an attractive option for patients concerned about memory impairment or procedural risks. For many patients, this profile makes rTMS the more tolerable first choice before escalating to ECT [2].

The introduction of newer biological treatments, such as esketamine, has further diversified therapeutic options for treatment-resistant depression [3]. In this context, rTMS is generally considered after optimization of pharmacological therapy, including dose adjustments, medication switches, and adjunctive strategies, as well as after an adequate trial of evidence-based psychotherapy, positioning it after these first-line approaches but before more invasive neuromodulatory interventions such as ECT [2,3]. Deciding when to introduce rTMS in this context is not straightforward. The number of failed antidepressant trials, while useful as a reference point, does not capture the full clinical picture--episode duration, symptom severity, comorbidities, prior treatment response, and patient preference all bear on that decision and should be considered alongside pharmacological history [3].

Clinical guidelines focus heavily on when to start rTMS, but say relatively little about what to do after a successful course--a gap that has real consequences for patients with recurrent depression. A substantial proportion of patients who respond to rTMS retain clinical benefit for several months without additional intervention, though a meaningful subset experiences symptom recurrence over time [8]. The question of how to sustain that benefit, including the role of maintenance protocols, is discussed in the section on stimulation protocols and technical considerations.

Neurobiological Mechanisms of rTMS in Depression

**Figure 1:**
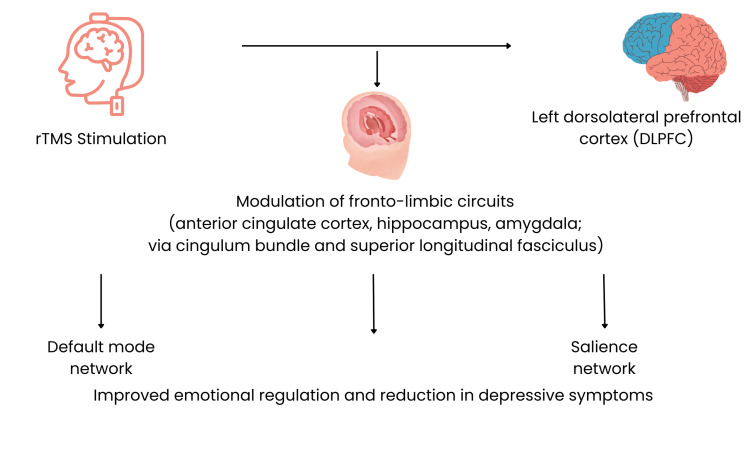
Mechanism of action of rTMS in major depressive disorder Repetitive transcranial magnetic stimulation (rTMS) targeting the left dorsolateral prefrontal cortex (DLPFC) modulates fronto-limbic circuits, including the anterior cingulate cortex, hippocampus, and amygdala, via white matter pathways such as the cingulum bundle and superior longitudinal fasciculus. This modulation influences large-scale brain networks, particularly the default mode network and salience network, leading to improved emotional regulation and a reduction in depressive symptoms. This figure represents a schematic model of proposed neurobiological mechanisms and does not depict empirically established pathways [9]. The image was created by the authors using Canva graphic design software (canva.com; Canva Pty Ltd., Sydney, Australia).

The therapeutic effects of rTMS in MDD are believed to arise from its ability to modulate neural circuits involved in emotional regulation rather than from isolated effects on a single cortical region. Most therapeutic protocols target the left dorsolateral prefrontal cortex (DLPFC), a region involved in cognitive control and the regulation of limbic activity [9]. Dysfunction within this region and its associated networks has been consistently implicated in the pathophysiology of depression. The proposed neurobiological mechanisms underlying rTMS are summarized in Figure 1.

At the synaptic level, the most widely accepted framework for understanding the lasting effects of rTMS holds that repeated magnetic stimulation induces changes in synaptic efficacy analogous to long-term potentiation and long-term depression. High-frequency stimulation above 5 Hz is generally associated with facilitatory effects that increase cortical excitability, while low-frequency stimulation at or below 1 Hz tends to produce inhibitory effects that decrease it [10]. These frequency-dependent effects are thought to involve changes in N-methyl-D-aspartate (NMDA) receptor activation and the regulation of postsynaptic intracellular calcium levels, which are established determinants of the direction of synaptic plasticity [10]. In practical terms, this is why high-frequency stimulation targets the left DLPFC while low-frequency protocols target the right--each working in opposite directions toward the same therapeutic goal, as outlined in current guidelines [11,12].

One of the principal neurobiological models proposes that rTMS exerts its antidepressant effects through the modulation of fronto-limbic circuits. Functional neuroimaging studies have demonstrated that stimulation of the DLPFC can influence activity in deeper structures involved in mood regulation, including the anterior cingulate cortex, amygdala, and hippocampus [13,14]. In particular, the subgenual anterior cingulate cortex has been repeatedly associated with treatment response in depression [14].

Beyond localized cortical effects, rTMS also appears to influence large-scale brain networks involved in emotional processing [15,16]. These include the default mode network, which is associated with self-referential thinking and rumination, and the salience network, which contributes to the detection and integration of emotionally relevant stimuli [15,16]. Altered interactions between these networks have been consistently observed in patients with MDD [15-17]. If rTMS does indeed shift how these networks interact, that shift may help explain why some patients report feeling less stuck in negative thought patterns after treatment - though this remains difficult to verify directly in clinical practice.

Structural connectivity also plays an important role in the propagation of rTMS effects across distributed neural circuits. White matter pathways linking prefrontal and limbic regions are believed to facilitate the transmission of stimulation-induced activity to deeper brain structures. At the functional level, the subgenual anterior cingulate cortex (sgACC) and its connections with both the default mode network and the salience network have received particular attention as potential mediators of how rTMS effects propagate from the DLPFC to deeper limbic structures [16]. Similarly, the superior longitudinal fasciculus provides structural connections between frontal and parietal regions that participate in cognitive control networks relevant to mood regulation.

Another pathway of interest is the uncinate fasciculus, which connects the orbitofrontal cortex with the anterior temporal lobe and the hippocampus. This tract has been implicated in emotional learning and reward processing, and alterations in its structural integrity have been associated with affective disorders [18]. Whether differences in white matter tract integrity help explain why some patients respond to rTMS and others do not is an open question that deserves more direct study.

What these findings collectively suggest is that the magnetic pulse at the scalp is only the starting point--the clinical effect unfolds across a network, not just beneath the coil.

Predictors of Treatment Response

Despite years of research, clinicians still cannot reliably predict which patients will respond to rTMS before treatment begins. Efficacy at the group level is not in doubt, but a substantial proportion of treated patients see little benefit, and no biomarker or clinical measure has yet proven reliable enough to identify them in advance [13,14]. Meta-analytic data suggest that response rates in controlled trials, typically defined as a 50% or greater reduction in depressive symptoms, hover around 29% for high-frequency rTMS in sham-controlled trials, assessed at the end of acute treatment in patients with MDD, meaning that roughly 70% of treated patients do not meet formal response criteria following a standard course [19]. Several candidate predictors have been identified in the research literature, but their clinical translation remains limited. Most findings derive from relatively small and heterogeneous samples, and substantial variability in stimulation protocols and outcome measures across studies makes cross-trial comparisons difficult [4,9].

Functional neuroimaging has generated some of the more studied, though not always consistent, findings in this area. Several studies have examined resting-state functional connectivity between the left DLPFC and the sgACC as a potential predictor of treatment outcome, with some reporting that greater anticorrelation between these regions at baseline is associated with better clinical outcomes following rTMS [13,14]. These findings align with neurobiological models proposing that rTMS exerts its antidepressant effects partly through downstream modulation of sgACC activity via fronto-limbic circuits [13,14]. However, this association has not held consistently across studies, and its reliability as a clinical predictor remains uncertain. Separately, Drysdale and colleagues identified distinct neurophysiological subtypes of depression, defined by patterns of resting-state connectivity across limbic and frontal regions, which showed differential responses to rTMS, with one subtype demonstrating markedly better outcomes than others [17]. These findings raise the possibility that connectivity-based biotyping could eventually contribute to more principled patient selection. In practice, most clinics that offer rTMS do not have access to resting-state fMRI, and even where it is available, the question of whether connectivity-based predictions hold up across different scanners and sites has not been settled.

Compared to neuroimaging, clinical variables are readily available at the time of referral and require no additional testing, making them a practical starting point for thinking about prognosis. Among the most consistently studied factors is the extent of prior pharmacological treatment resistance: patients who have failed more than two adequate antidepressant trials show lower odds of achieving remission following rTMS than those with fewer prior failures, a finding observed both in meta-analytic data and in large randomized trial analyses [4,20]. This does not mean that more treatment-resistant patients should be excluded from rTMS--evidence supports its use across this spectrum--but it does suggest that prognostic expectations should be adjusted based on the degree of prior treatment failure [3]. Regarding baseline symptom severity, available data indicate that patients with mild-to-moderate depression at baseline reach remission at higher rates than those presenting with severe depression, with one study reporting remission rates of 60% versus 19%, respectively [21]. Episode duration has also been examined as a predictor, with longer current episodes associated with lower odds of response, though this finding requires replication in larger and more methodologically uniform samples [3]. At the symptom profile level, higher baseline anxiety severity has been independently associated with lower remission rates following rTMS [20], while the presence of psychotic features is generally considered a clinical context in which rTMS as a standalone intervention is not appropriate [2]. Age also matters. Older adults tend to respond less robustly to rTMS than younger patients, a finding that has held up in trials designed specifically for late-life depression [22].

The pharmacological context in which rTMS is delivered is a clinically relevant variable that remains inadequately studied in the existing literature. Among the medications of greatest interest, benzodiazepines have received particular attention. A study examining concomitant medication use in 181 patients undergoing rTMS found that benzodiazepine use was associated with less clinical improvement early in the treatment course, with lower response rates at six weeks compared to non-users [23]. This association was also observed in trajectory analyses from the THREE-D trial, where benzodiazepine users were underrepresented in the rapid-response group [24]. However, these findings are not uniform across studies, and the clinical significance of this interaction has not been firmly established. Current guidelines note that rTMS can be administered in the presence or absence of concurrent antidepressant or psychotropic medication, addressing concomitant drug use primarily from a safety rather than an efficacy standpoint [2]. No prospective trial has yet tested whether tapering benzodiazepines before starting rTMS actually improves outcomes--a straightforward clinical question that remains unanswered.

Beyond clinical and neuroimaging variables, research has explored neurophysiological and genetic markers as potential tools for refining patient selection. Among genetic factors, the Val66Met polymorphism in the BDNF gene, which modulates the activity-dependent secretion of BDNF and thereby influences the capacity for synaptic plasticity, has been studied in the context of TMS-induced cortical responses, with Met allele carriers showing reduced or absent facilitatory responses to plasticity-inducing TMS protocols compared with Val/Val individuals [25]. At the neurophysiological level, EEG-derived measures have attracted growing interest as accessible and cost-effective candidate predictors. Frontal alpha asymmetry measured at baseline has been associated with positive response to TBS-rTMS in treatment-resistant depression, with responders showing lower asymmetry values prior to treatment, though replication in larger samples is needed before this measure can be considered a reliable clinical tool [26]. These converging lines of evidence point toward a future in which patient selection for rTMS is guided by multiple data sources, an approach increasingly described as precision psychiatry. Getting there will require the kind of large, multicenter, methodologically consistent research that the field has not yet produced.

Stimulation Protocols and Technical Considerations

The clinical effects of rTMS depend not only on the cortical target but also on the stimulation parameters used during treatment. Several stimulation protocols have been developed over time for the management of MDD, most of which focus on stimulation of the left dorsolateral prefrontal cortex [2,9].

High-frequency rTMS delivered to the left dorsolateral prefrontal cortex remains one of the most commonly used therapeutic approaches in clinical practice. Frequencies around 10 Hz are typically applied to increase cortical excitability in prefrontal regions that tend to be functionally hypoactive in patients with depression [2]. Treatment is usually administered five days per week over several weeks, with each session consisting of multiple trains of stimulation delivered at an intensity defined as a percentage of the patient’s motor threshold [2].

A second well-established protocol targets the right DLPFC using low-frequency stimulation, typically delivered at 1 Hz. The neurobiological rationale for this approach differs from that of high-frequency left-sided stimulation: rather than increasing excitability in a hypoactive left prefrontal cortex, low-frequency right-sided stimulation is intended to reduce activity in a presumably hyperactive right prefrontal cortex, with the goal of restoring interhemispheric balance [11]. Meta-analytic evidence supports its antidepressant efficacy, with response rates of approximately 38% in active treatment groups compared to 15% in sham controls, and a number needed to treat of 5 for both response and remission [27]. Direct comparisons with high-frequency left-sided protocols have found broadly similar clinical efficacy between the two approaches, with response rates of approximately 45% for high-frequency and 41% for low-frequency stimulation across randomized trials [12]. From a safety standpoint, the lower seizure risk associated with 1 Hz stimulation makes this protocol worth considering in patients with a history of epilepsy or other factors that lower the convulsive threshold [11].

Alternative stimulation strategies have also been developed to improve treatment efficiency and reduce session duration. One of the most widely studied approaches is intermittent theta burst stimulation (iTBS), a patterned protocol designed to reproduce endogenous theta rhythms observed in the brain. Compared with conventional high-frequency rTMS, iTBS can be delivered in considerably shorter sessions while maintaining similar clinical effectiveness [22]. Shorter sessions may improve patient adherence to treatment and may help bring neuromodulation into routine clinical practice.

Beyond conventional surface coil approaches, a distinct technical variant known as deep transcranial magnetic stimulation (dTMS) has been developed using specially designed H-coils, which are intended to reach deeper and broader cortical regions than standard figure-of-eight coils. The antidepressant efficacy of the H1-coil was evaluated in a prospective, double-blind, multicenter randomized controlled trial involving 212 patients with MDD who had not responded to one to four prior antidepressant trials. This study provided the clinical evidence base that supported regulatory clearance of the Brainsway deep TMS system by the United States Food and Drug Administration [28]. A separate line of development has focused on compressing the treatment timeline itself rather than expanding the stimulation volume. The most extensively studied of these is the Stanford Neuromodulation Therapy, previously known as SAINT, which delivers multiple iTBS sessions per day over five days using individualized fMRI-guided targeting. In a double-blind, sham-controlled randomized trial, active treatment was associated with a remission rate of 78% compared with 13% in the sham group [29]. The remission rates reported are striking, but the trial was small and enrolled carefully selected outpatients--a very different population from the patients seen in most psychiatric services. Replication in larger, more representative samples is needed before SAINT can be recommended broadly.

Accurate localization of the stimulation target is another important technical consideration. Earlier approaches relied on simple scalp-based measurements, such as the “5-cm rule,” to estimate the position of the dorsolateral prefrontal cortex relative to the motor cortex. More recently, neuronavigation systems based on structural or functional neuroimaging have been introduced to improve targeting precision and better align stimulation with relevant cortical networks [2].

Most of the evidence base for rTMS focuses on the acute treatment phase. What happens to patients after they respond, and how to keep them well, has received far less attention than it deserves. Relapse rates following acute rTMS can reach up to 70% in patients who initially responded to treatment, with the risk appearing most pronounced around five months after treatment completion, based on systematic review data [30]. Maintenance rTMS, defined as periodic sessions administered at reduced frequency following the acute phase, has been proposed as one approach to sustaining the antidepressant benefit in patients who have responded. A recent randomized clinical trial comparing maintenance low-frequency rTMS with lithium pharmacotherapy in 75 patients with treatment-resistant depression found comparable relapse prevention efficacy between the two approaches, with seven relapses in each group, while the rTMS group experienced significantly fewer adverse events [31]. Available evidence also suggests that administering two or fewer sessions per month may be insufficient to sustain an antidepressant effect, with more frequent protocols appearing more effective at reducing relapse risk [30]. None of the major international guidelines currently recommends a specific maintenance protocol after acute rTMS. How often sessions should be given, for how long, and for which patients--these questions do not yet have clear answers.

The choice of stimulation protocol, treatment intensity, and targeting method can influence clinical outcomes following rTMS therapy. Small improvements in protocol optimization can matter a great deal to patients who have already exhausted standard options.

Safety and Adverse Effects

rTMS is generally considered a safe and well-tolerated treatment for MDD. Compared with other neuromodulation therapies, particularly ECT, rTMS is associated with a more favorable adverse-effect profile and does not require anesthesia or systemic sedation [2].

The most commonly reported adverse effects are mild and transient. Patients frequently describe scalp discomfort or headache during or shortly after stimulation sessions, which typically diminish as treatment progresses and rarely require discontinuation of therapy [2]. Although uncommon, treatment-emergent hypomanic or manic symptoms have been reported following rTMS, particularly in patients with a prior history of bipolar disorder or mood instability. Clinicians should monitor for early signs, such as agitation, irritability, and insomnia, during the treatment course [2]. Anxiety and agitation have also been noted as transient adverse effects, typically mild and self-limiting [2]. Local muscle twitching in facial or scalp muscles may also occur during stimulation, but is usually well-tolerated.

It is also important to note that rTMS is contraindicated in patients with ferromagnetic implants near the stimulation site, cochlear implants, implanted neurostimulators, or cardiac pacemakers, as the magnetic field may interfere with these devices. A history of epilepsy or unprovoked seizures represents an additional contraindication that requires careful evaluation prior to initiating treatment [11].

Serious adverse events are rare. The most notable potential complication is seizure induction; however, when stimulation parameters follow established safety guidelines, the risk remains extremely low [2,11]. Careful patient selection and adherence to recommended stimulation protocols further reduce this risk.

Another advantage of rTMS compared with ECT is the minimal impact on cognitive function. Unlike ECT, which can be associated with transient memory impairment, rTMS has not been consistently linked to clinically significant cognitive adverse effects [7]. In some studies, modest improvements in certain cognitive domains have even been observed during treatment.

Taken as a whole, rTMS is well-tolerated: a fact that matters enormously for patients who are already managing a debilitating condition and may be reluctant to accept treatments with significant side effect burdens.

Limitations

This review has several limitations worth noting. As a narrative review, the selection of studies relied on clinical judgment rather than a formal systematic protocol, so some relevant studies may not have been included. We did not apply PRISMA criteria or conduct a formal quality assessment of individual studies. The literature on rTMS is also quite heterogeneous. Studies vary considerably in stimulation parameters, patient characteristics, and how outcomes are measured, which makes direct comparisons difficult. Some of the neuroimaging studies cited, particularly those looking at predictors of response, involved relatively small samples, and their findings should be interpreted with that in mind. Publication bias is another factor that cannot be ruled out. These limitations notwithstanding, we hope this review gives clinicians a practical sense of where rTMS stands today--what the evidence supports, where it falls short, and what questions still need answering.

## Conclusions

One of the clearest messages from the literature review is that rTMS does not work in isolation at the cortical surface; its effects propagate through fronto-limbic circuits and large-scale brain networks that are known to be dysregulated in depression. Understanding this has changed how the field thinks about the treatment, moving it from a largely empirical procedure toward one with a growing mechanistic rationale. As discussed earlier, the efficacy of rTMS at the group level is well-established. What remains uncertain is how to translate that group-level evidence into reliable predictions for individual patients. In current treatment algorithms, rTMS occupies a defined yet still evolving role. Although guidelines recommend its use after inadequate response to pharmacological therapy, the optimal timing of its introduction, as well as its integration with pharmacological and psychotherapeutic strategies, remains uncertain. Esketamine adds another layer of complexity to this decision--two rapid-acting options now exist, and clinicians have little evidence to guide how they should be sequenced or combined. On the practical side, theta burst protocols have made rTMS faster without sacrificing efficacy, a genuine improvement for both patients and clinics. The bigger problem is that outcomes still vary widely between individuals. Some patients remit; others see no benefit. Without reliable predictors, clinicians largely cannot know in advance which outcome to expect. Neuroimaging-based biomarkers, particularly measures of resting-state connectivity, have shown early promise as predictors of response, but none is ready for routine clinical use. Most findings have not been replicated at the scale required to change practice.

rTMS has earned its place in the treatment of major depressive disorder. The question of whether rTMS works--at the group level, in carefully selected populations--is largely settled. What the field now needs to answer is who it works for, when to use it, and how to sustain its benefits over time--questions that are harder than demonstrating efficacy and ultimately more important for the patients who need it most.
